# H4 Histamine Receptors Mediate Cell Cycle Arrest in Growth Factor-Induced Murine and Human Hematopoietic Progenitor Cells

**DOI:** 10.1371/journal.pone.0006504

**Published:** 2009-08-07

**Authors:** Anne-France Petit-Bertron, François Machavoine, Marie Paule Defresne, Michel Gillard, Pierre Chatelain, Prakash Mistry, Elke Schneider, Michel Dy

**Affiliations:** 1 Université Paris Descartes, Faculté de Médecine, CNRS UMR8147, Hôpital Necker, Paris, France; 2 Department of Cytology and Histology, University of Liège, Liège, Belgium; 3 UCB S.A. Pharma, Chemin du Friest, Braine-l’Alleud, Belgium; 4 UCB Celltech, Slough, United Kingdom; Universidade Federal do Rio de Janeiro (UFRJ), Instituto de Biofísica da UFRJ, Brazil

## Abstract

The most recently characterized H4 histamine receptor (H4R) is expressed preferentially in the bone marrow, raising the question of its role during hematopoiesis. Here we show that both murine and human progenitor cell populations express this receptor subtype on transcriptional and protein levels and respond to its agonists by reduced growth factor-induced cell cycle progression that leads to decreased myeloid, erythroid and lymphoid colony formation. H4R activation prevents the induction of cell cycle genes through a cAMP/PKA-dependent pathway that is not associated with apoptosis. It is mediated specifically through H4R signaling since gene silencing or treatment with selective antagonists restores normal cell cycle progression. The arrest of growth factor-induced G1/S transition protects murine and human progenitor cells from the toxicity of the cell cycle-dependent anticancer drug Ara-C *in vitro* and reduces aplasia in a murine model of chemotherapy. This first evidence for functional H4R expression in hematopoietic progenitors opens new therapeutic perspectives for alleviating hematotoxic side effects of antineoplastic drugs.

## Introduction

Histamine is one of the most versatile biogenic amines with pleiotropic activities, including regulatory functions during the immune response and hematopoiesis [1]–[3]. This functional diversity results from the variety of its modes of intervention through extra- and intracellular binding sites and specific receptors, triggering different signal transduction pathways [4]–[6]. The final outcome of these interactions is quite complex, as it depends on how receptors are distributed on target cells, according to their microenvironment and stage of development [7], [8].

Even though the most recently discovered H4R is mainly expressed in the bone marrow (BM) [9], its potential role during hematopoiesis has not been addressed. To date, its most clearly established functions consist in recruitment and activation of hematopoietic cells involved in inflammatory responses, such as eosinophils, mast cells, neutrophils and dendritic cells [10]–[14]. Because of these activities, together with H4R-induced IL-16 production by CD8 cells [15] and alleviation of experimental allergic asthma in H4R-deficient mice [16], this receptor is considered a potential pharmacological target for anti-inflammatory therapy [17].

Histamine has been implicated in the regulation of hematopoietic progenitor cells by several studies, including those of J. W. Byron and our own [18], [19]. These activities have been ascribed to H1 and H2 histamine receptors, the only subtypes known at the time. The discovery of an additional H4R, together with its predominant expression in the bone marrow, prompted us to reassess this issue. Here we report that the H4R is preferentially expressed and functional in progenitor-enriched murine and human hematopoietic cells, as it mediates a reversible cAMP/PKA-dependent cell cycle arrest that causes reduced proliferation and colony formation in methylcellulose. Based on the notion that quiescence protects clonogenic cells from growth-dependent cytotoxicity, we investigated whether H4R activation could become instrumental in a clinical setting to prevent myeloablation in a murine model of chemotherapy.

## Results

### Functional H4R expression in murine hematopoietic progenitor cells

**Figure 1:**
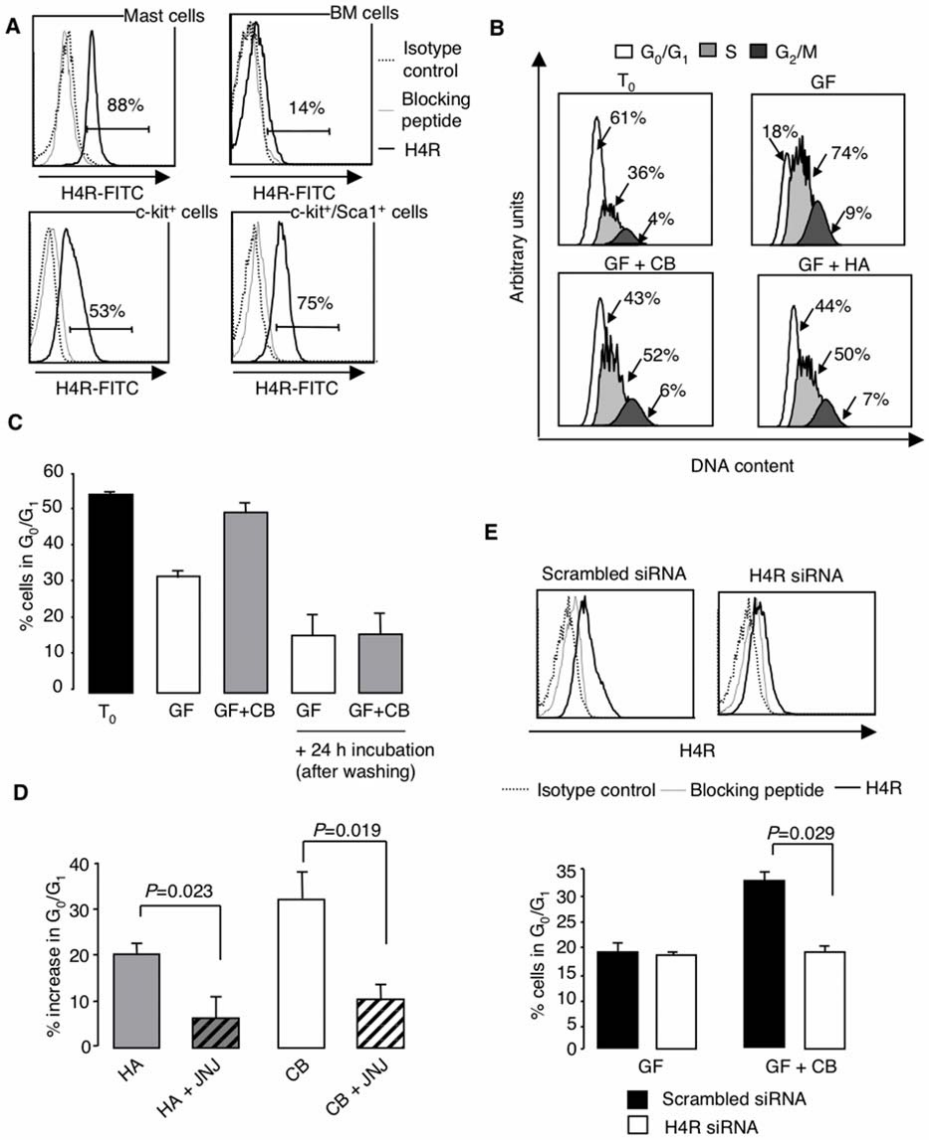
Functional H4R expression in murine hematopoietic progenitor cells. (A) Staining of total, sorted c-kit^+^ and c-kit^+^Sca1^+^ BM cells with anti-H4R antibody compared with irrelevant isotype control and anti-H4R antibody saturated with blocking peptide. BM-derived murine mast cells served as a positive control. (B) Cell cycle arrest in sorted progenitor-enriched c-kit^+^ BM cells after a 2-h incubation in StemSpan medium supplemented with growth factor cocktail (GF), with or without histamine or CB at a concentration of 10^−5^ M. The cell cycle status was analyzed after staining with Vybrant DyeCycle Violet (VDV) as compared with freshly isolated cells. (C) Reversal of H4R-induced cell cycle arrest. Sorted c-kit^+^ cells were incubated for 3 days with or without CB in StemSpan medium supplemented with growth factor cocktail. The cell cycle status was then assessed before and after further incubation of extensively washed cells with growth factors for 24 h. Data are means±SD from 2 experiments. Unstained apoptotic cells were not detected. (D) Recovery of normal cell cycling in the presence of a selective H4R antagonist. Sorted c-kit^+^ BMC were incubated for 3 days with growth factors (GF) alone or together with histamine (HA) or CB at a concentration of 10^−5^ M, with or without prior exposure to JNJ 7777120 (10^−5^ M). Means±SEM from 3 experiments. (E) H4R silencing in c-kit^+^ BM cells. H4R expression was evaluated 24 h after transfection (>90% efficiency). The effect of gene silencing on CB-induced cell cycle arrest was assessed following overnight incubation. Means±SEM from 3 experiments.

We assessed the expression of the H4R in total and progenitor-enriched bone marrow (BM) populations by staining with specific antibodies. As shown in **Fig. 1A**, the proportion of positive cells increased from total BM to progenitor-enriched c-kit^+^ and more primitive c-kit^+^Sca1^+^ cells, which proved that the receptor is mainly expressed in the immature compartment. Murine bone marrow-derived mast cells are shown as a positive control.

We evaluated the function of the H4R, by examining the effect of histamine, its natural ligand, and clobenpropit (CB), one of its most potent agonists, on the cell cycle status of sorted c-kit^+^ BM cells, which comprise a heterogenous population of progenitors at various differentiation stages. As shown in **Fig. 1B**, these cells are mostly quiescent at t_0_, but enter the cell cycle in response to a growth factor cocktail composed of IL-3, IL-6 and SCF. Both histamine and CB inhibited growth factor-induced cell cycle progression at an optimal dose of 10^−5^ M (dose-response curve in **Figure S1**), as illustrated by the accumulation of cells in stage G_0_/G_1_ and decrease in G_2_/S. The cell cycle arrest persisted after 3 days in the presence of CB, but could by reversed upon its removal by a 24-h exposure to growth factor cocktail (**Fig. 1C**). Apoptosis was not induced in these conditions, as assessed by Annexin-V/PI staining illustrated by the dot plots of a typical experiment in **Figure S2**.

Though initially developed as an H3R antagonist, CB is H4R-specific in BM cells, which do not express the H3R [5]. Pretreatment with the selective H4R antagonist JNJ7777120 [20] before exposure to histamine or CB (**Fig. 1D**) prevented the cell cycle arrest, providing an additional argument in favor of H4R specificity. This was confirmed by a similar effect of the antagonist JNJ10191585 and the reverse agonist thioperamide (**Figure S3**). Finally, growth factor-induced cell cycle progression was restored in progenitor-enriched ckit^+^ BM cells transfected with siRNA to knock down H4R expression before exposure to CB (**Fig. 1E**). This treatment did effectively diminish H4R expression on protein levels, proving the specificity of the effect.

### H4R-mediated inhibition of cell proliferation and/or differentiation of clonogenic progenitors

**Figure 2:**
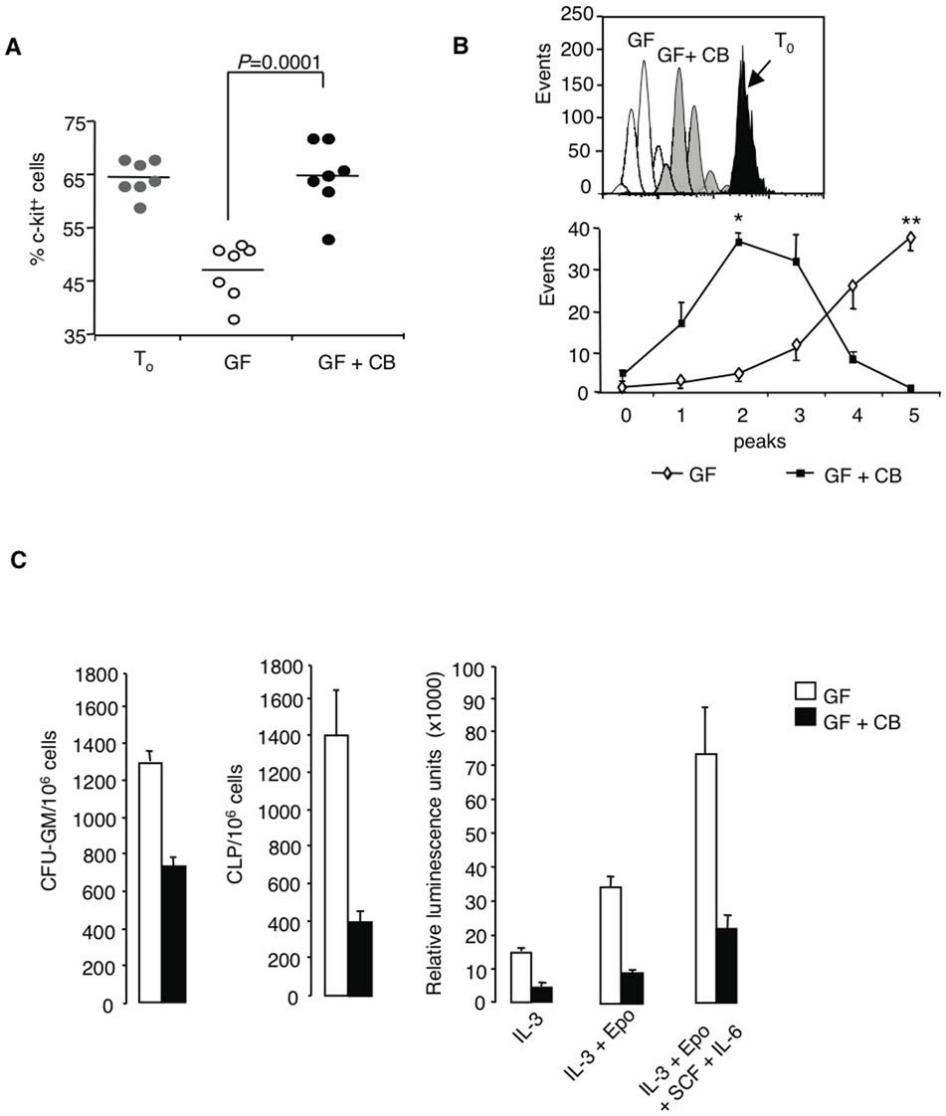
H4R-mediated inhibition of clonogenic cell proliferation and/or differentiation. (A) Maintenance of c-kit expression by Lin^-^ BMC incubated for 3 days in growth factor cocktail (GF) together with CB (10^−5^ M), as compared with c-kit^+^ cells freshly sorted or cultured in GF alone. (B) Decreased number of cell divisions upon H4R stimulation. After staining with CFSE, Lin^−^ BM cells were incubated for 3 days in growth factor cocktail with or without CB. CFSE fluorescence intensity was analyzed among gated c-kit^+^ cells. (Means±SEM from 6 experiments. *P = 0.013 **P = 0.0022). A typical FACS profile is shown. (C) Inhibition of colony formation from total BM cells in the presence of CB. Cells were seeded into MethoCult supplemented with 1 ng/ml of mrIL-3 to generate granulocyte/macrophage colonies or with SCF (100 ng/ml)+IL-7 (10 ng/ml+Flt-3-L (20 ng/ml) to reveal common lymphoid progenitors (CLP). CB was added at a concentration of 10^−5^ M and colonies were scored after 7 days of culture. Means±SEM. from 3 experiments. The right panel depicts the results obtained with the HALO^TM^-Hemogenix assay based on luminescence output in response to growth factor cocktails revealing CFU-GM, CFU-GM+BFU-E and CFU-GM+BFU-E+CFU-GEMM. Means±SEM from quadruplicate determinations.

The inhibition of growth factor-induced G1/S transition in the presence of CB led to reduced proliferation and/or differentiation, as revealed 1) by the maintenance of c-kit expression among progenitor-enriched cells depleted for mature BM components (Lin^-^) after a 3-day culture in the presence of CB (**Fig. 2A**) and 2) by a reduced number of cell divisions measured by CFSE tracking (**Fig. 2B**), relative to growth factor-stimulated controls. H4R activation inhibited likewise the proliferation of more primitive sorted c-kit^+^Sca1^+^ cells (78±5% inhibition in response to CB after 3 days of culture with growth factor cocktail; mean±SEM; n = 3), in agreement with their H4R expression (**Fig. 1A**). As shown in **Fig. 2C**, it caused a marked decrease in both myeloid (CFU-GM) and lymphoid (CLP) colony formation in methylcellulose. A comparable inhibition occurred in the HALO-Hemogenix luminescence assay, set up with different growth factor cocktails to reveal proliferation of progenitors with distinct differentiation potentials (CFU-C, CFU-C+BFU-E and CFU-C+BFU-E+CFU-GEMM) (**Fig. 2C**). Once again, the H4R antagonist JNJ7777120 reversed the inhibition of CB on IL-3-induced colony formation (10.5±5.1% in the presence of JNJ versus 55.1±10.3% in response to CB alone; n = 3).

### Impaired expression of cell cycle proteins through cAMP/PKA-dependent signaling triggered by H4R engagement

**Figure 3:**
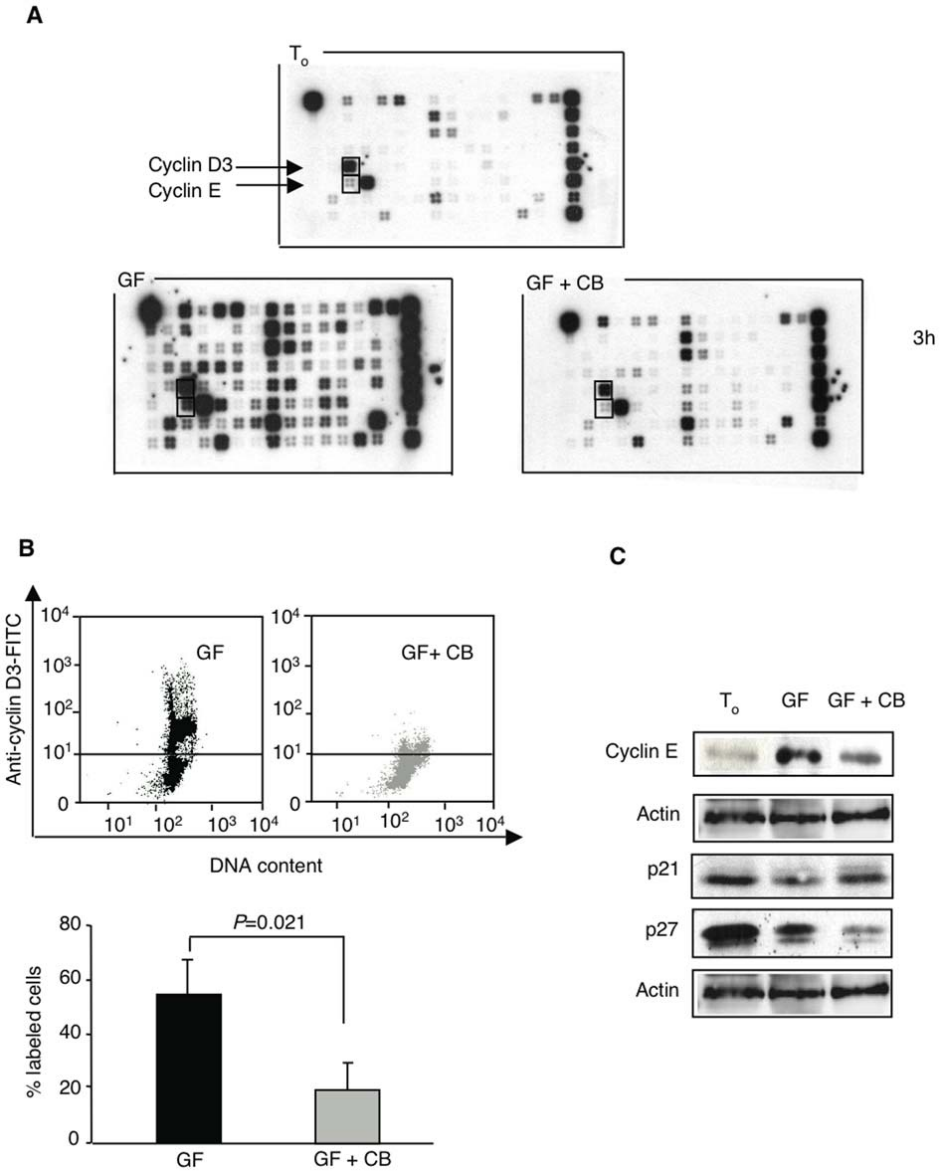
H4R activation impairs the expression of cell cycle proteins. (A) The effect of 10^−5^ M CB on the expression of cell cycle genes was assessed in freshly isolated c-kit^+^ BM cells and after a 3-h exposure to growth factor cocktail (GF) with or without CB, using a cell cycle Oligo GEArray. Fold decrease upon exposure to CB: 35 for cyclin D3: and: 54 for cyclin E. (B) Decreased intracellular staining with FITC-conjugated anti-cyclin D3 antibody after an overnight incubation of sorted c-kit^+^ cells in the presence of CB, as compared with growth factors alone. Data represent a typical dot plot and means±SEM from 3 experiments. (C) Western blot analysis in whole-cell extracts before and after a 5-h incubation of sorted c-kit^+^ cells with growth factors alone or together with CB.

We examined the impact of H4R activation on the expression of genes encoding cell cycle proteins. As shown in **Fig. 3A**, the overall transcription of growth factor-induced cell cycle genes (oligo GEArray® 0MM-020) decreased strikingly upon exposure to CB, almost to the level of freshly isolated cells. Further analysis of cyclins expressed typically during G1/S transition [21] established that both cyclin D3 and E were markedly decreased in response to CB, as demonstrated by FACS and Western blot analysis (**Fig. 3B and C**, respectively). In addition, the H4R agonist prevented growth factor-induced downregulation of the cyclin-dependent kinase inhibitor p21^Cip1/Waf1^, while p27^Kip1^ expression was decreased (**Fig. 3C**).

**Figure 4:**
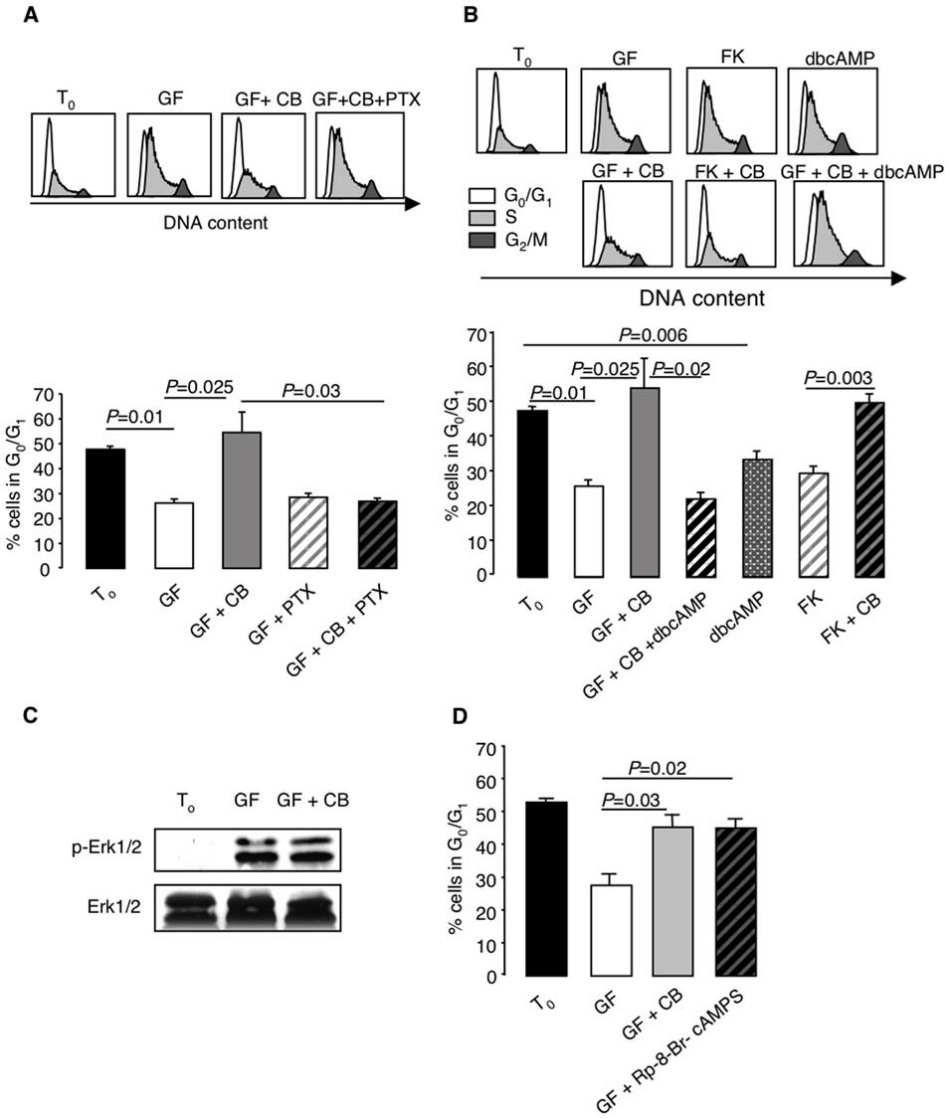
CB-induced cell cycle arrest is mediated through G_i/o_ proteins and cAMP/PKA-dependent signaling. (A) Sorted c-kit^+^ BM cells were exposed for 2 h to growth factor cocktail with or without CB and/or PTX (100 ng/ml). The cell cycle status was analyzed after staining with Vybrant DyeCycle Violet (VDV), in comparison with freshly isolated cells. Means±SEM from 3 experiments. (B) Critical role of cAMP in CB-induced cell cycle arrest. Sorted c-kit^+^ BM cells were incubated for 2 h in StemSpan medium with growth factor cocktail or forskolin (FK; 10^−5^ M) with or without CB, dbcAMP alone or together with growth factors and CB. The cell cycle status was assessed by Vybrant DyeCycle Violet (VDV) staining. Data represent typical cell cycle profiles and means±SEM from 3 experiments. (C) Western blot analysis of Erk expression in freshly isolated c-kit^+^ cells and after 5 h of incubation with growth factor cocktail (GF) with or without CB. (D) The PKA inhibitor Rp-8-Br-cAMPS mimics the cell cycle arrest induced by CB in sorted c-kit^+^ BM cells stimulated with growth factor cocktail. The cell cycle status was evaluated by VDV staining after a 2-h incubation.

CB did no longer inhibit cell cycling in progenitor-enriched BM cells treated with *Bordetella pertussis* toxin (PTX), which confirmed the involvement of G_i/o_ protein coupling in the initiation of the signal transduction pathway (**Fig. 4A**). The decrease of intracellular cAMP levels is critical for cell cycle arrest that is counteracted by adding exogenous dbcAMP, a cell-permeable analogue of this signal carrier (**Fig. 4B**). In further support of this mechanism of action, CB inhibited cell cycle progression induced by forskolin, commonly used to raise intracellular cAMP levels. As shown in **Fig. 4C**, it did not affect the expression of Erk, conversely to other models in which a crosstalk between cAMP and the ERK/MAPK pathway has been described [22]. The PKA/CREB pathway is the most likely candidate for downstream signaling [23], given that the PKA inhibitor Rp-8-Br-cAMPS mimicked the effect of CB (**Fig. 4D**).

### H4R-mediated *in vitro* and *in vivo* protection of murine hematopoietic progenitor cells against the toxicity of anti-cancer drugs

**Figure 5:**
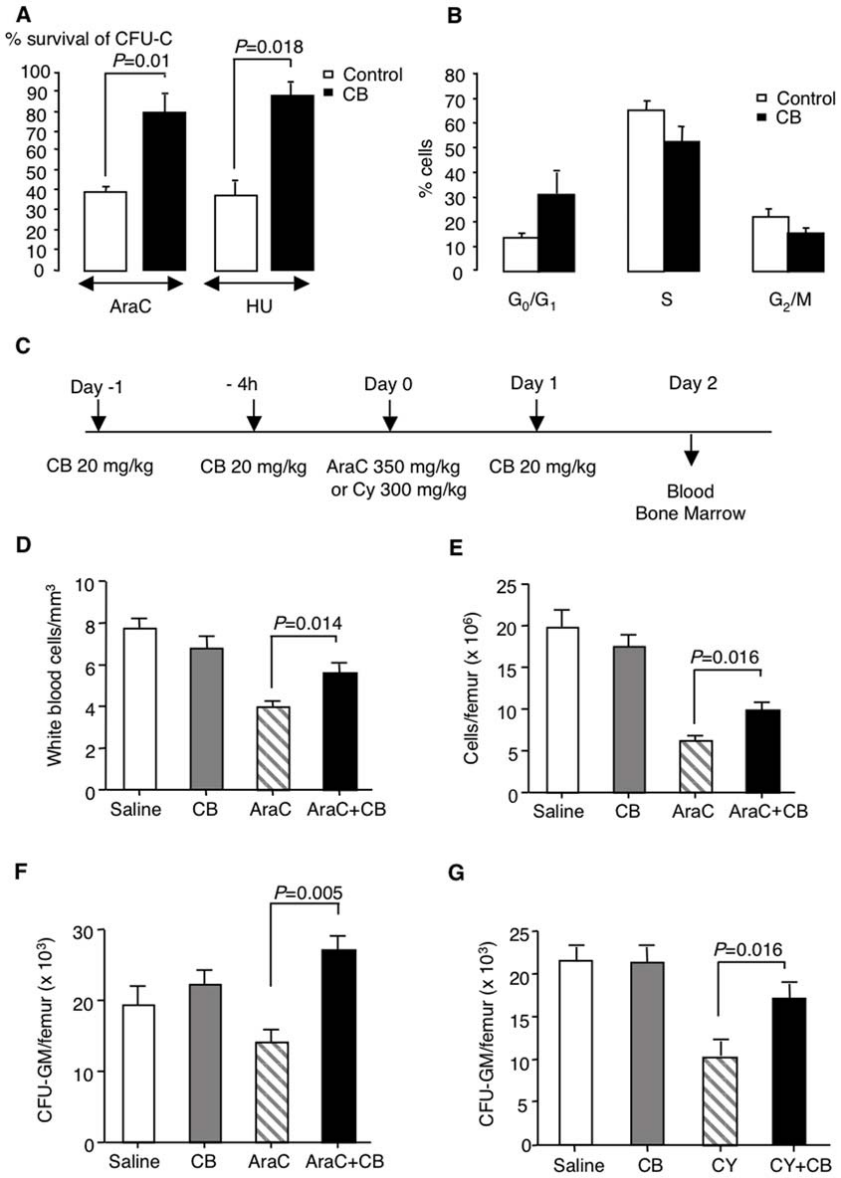
H4R-induced cell cycle arrest in murine hematopoietic progenitors *in vivo* and protection against the cytotoxicity of cell cycle-dependent chemotherapeutic compounds. (A) BM cells were pretreated for 24 h with CB, before exposure to AraC (10 µg/ml), or hydroxy-urea (HU; 10^−3^ M). After a 1-h incubation, cells were extensively washed and plated in methylcellulose in the presence of IL-3 (1 ng/ml). 7 days later colonies were scored and survival with or without CB was evaluated relative to saline controls. (B) BM cells were recovered 4 h after the last of three injections of CB or saline (30 mg/kg each). The cell cycle status of gated Lin^−^ cells was evaluated *in vitro* after exposure to growth factors overnight (means±SEM; n = 3). (C) Injection schedule of CB in AraC- or cyclophosphamide-treated mice. (D-G) Total WB and BM cells were counted 2 days after injection of AraC in mice having received 3 i.p. injections of CB (20 mg/kg each) or saline, as compared with CB alone, Means±SEM from 8 individual mice. CFU-GM were evaluated in the BM of mice having received AraC or cyclophosphamide 2 days before sacrifice. Data are means±SEM. n = 8 and n = 2 in the AraC and cyclophosphamide series.

We argued that H4R-induced cell cycle arrest, which is reversible and not associated with apoptosis, might provide a strategy for myeloprotection. To test this assumption *in vitro*, we pretreated murine BM cells with or without CB, before stimulating their entry into cell cycle in response to growth factors, followed by exposure to the cell cycle-dependent anti-cancer drugs AraC or hydroxy-urea (HU). We then assessed their clonogenic potential in the methylcellulose colony-forming assay, as shown in **Fig. 5A**. We found that this treatment protected a substantial proportion of clonogenic progenitors, which opened the way for *in vivo* experiments, to verify whether cell cycle arrest occurred in mice having received repeat injections of CB. As shown in **Fig. 5B**, this turned out to be the case since the proportion of progenitor-enriched c-kit^+^ BM cells in phase G_0_/G_1_ of the cell cycle was significantly higher among cells recovered from mice having received CB before stimulation with growth factor cocktail than among their saline-injected counterpart.

We assessed myeloprotection through H4R activation in a model of cell cycle-dependent chemotherapy, following the schedule represented in **Fig. 5C**. At day 2 post AraC CB maintained significantly higher BM and white blood cell counts relative to AraC alone (**Fig. 5D and E**). This was likewise true for clonogenic cells evaluated in the methylcellulose assay (**Fig. 5F**). Treatment with CB alone had no significant effect on either of these parameters (**Fig. 5D-F**) and had no other adverse consequences. Following the same injection schedule, CB provided also a partial protection of medullary colony-forming cells 2 days post treatment of mice with the antineoplastic drug cyclophosphamide (300 mg/kg), a mobilizing agent that is only partially cell cycle-dependent (**Fig. 5E**).

### H4R-induced cell cycle arrest in human hematopoietic progenitor cells

**Figure 6:**
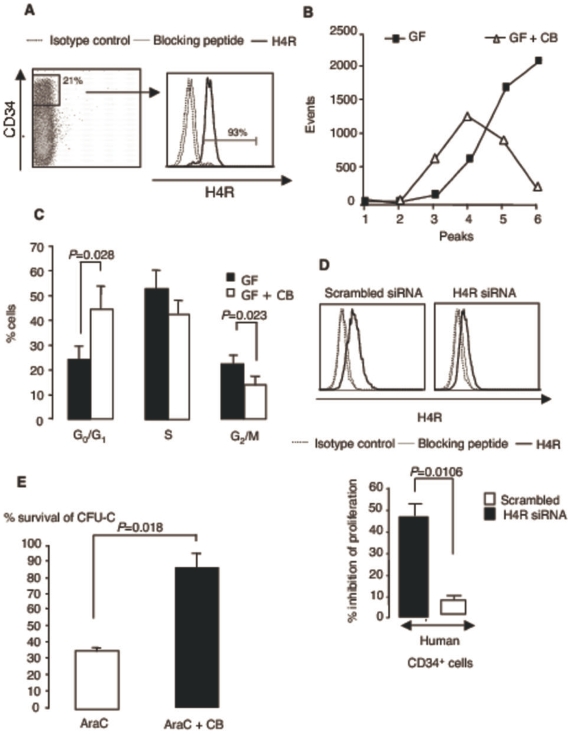
H4R activation induces inhibition of cell cycle progression in human progenitor cells. (A) H4R expression in gated CD34^+^ cells sorted from cord blood after a 3-day expansion in StemSpan medium with growth factor cocktail. (B) Tracking of cell divisions by CFSE staining. After expansion for 3 days, CD34^+^ were stained with CFSE and incubated for further 3 days in StemSpan medium supplemented with growth factor cocktail with or without CB. They were then labeled with PE-conjugated CD34 mAb and CFSE fluorescence intensity was measured in gated CD34^+^ cells. Data represent a typical experiment out of 2. (C) Cell cycle analysis. Cells were prepared as above, followed by staining with FITC-conjugated anti-CD34 mAb and PI. (Means±SEM; n = 3. (D) H4R silencing in CD34^+^ cells. H4R expression was evaluated 24 h after transfection (>90% efficiency) with silencing and scrambled RNA. The effect of gene silencing on CB-induced inhibition of proliferation was assessed after a further 3-day incubation when cells were counted and viability was assessed by trypan blue exclusion. Data represent means±SD from 2 experiments. (E) Protection of human clonogenic cells against the toxicity of AraC. CD34^+^ cells were pretreated for 24 h with CB, before exposure to AraC (10 µg/ml). After a 1-h incubation, cells were extensively washed and plated in methylcellulose supplemented with growth factor cocktail. 14 days later colonies were scored and survival with or without CB was evaluated relative to saline controls. (Means±SEM; n = 3).

We addressed the question whether the H4R was also expressed and functional in human hematopoietic progenitor cells. As shown in **Fig. 6A**, the most immature CD34^high^ cells isolated from cord blood and expanded for 3 days in growth factor cocktail were largely H4R-positive. Furthermore, their proliferation in response to the growth factor cocktail was clearly inhibited upon exposure to the agonist CB (3.8±1.4×10^6^ cells in response to growth factor cocktail alone versus 1.8±0.9×10^6^ in the presence of CB after 3 days of culture starting from 10^6^ cells; means±SEM; n = 5), in accordance with an induction of the cell cycle arrest. Indeed, H4R activation decreased the number of cell divisions, evaluated by CFSE staining (**Fig. 6B**) and slowed down cell cycle progression by increasing the percentage of cells in G_0_/G_1_ (**Fig. 6C**). Cell viability, determined by trypan blue exclusion and FACS analysis was not impaired in these conditions. As in the murine model, H4R specificity was established by gene silencing that abolished most of the anti-proliferative effect of CB on CD34^+^ cells stimulated with growth factor cocktail (**Fig. 6D**). A similar restoration of cell growth occurred after receptor blockade by JNJ7777120 (data not shown). Finally, a 24-h pretreatment with CB before exposure to AraC was clearly protective, supporting potential applications in a clinical setting (**Fig. 6E**).

### H4R expression and function in cancer cell lines

**Figure 7:**
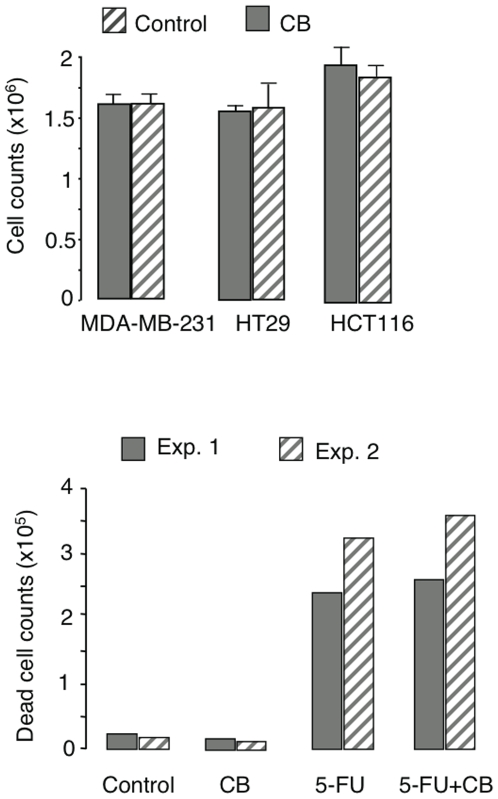
Malignant cell proliferation and sensitivity to 5-FU are not affected by C. (A) Proliferation of colon and mammary gland carcinoma cells was assessed after 3 days of culture starting from 2×10^5^ cells in 1 ml. Cells were detached by trypsin digestion and counted after staining with trypan blue. Data are means±SEM from three distinct experiments. (B) HCT116 carcinoma cells set up at a concentration of 10^5^/ml were pretreated 24 h with CB (10^−5^ M) before adding 5-FU (Sigma) at a concentration of 10 µg/ml for 48 h. 2 typical experiments are shown.

It is generally acknowledged the H4R expression occurs mainly in cells of hematopoietic origin. Nevertheless, the receptor has been reported in two well-known human colon cancer cell lines, HT29 and HCT116, and the mammary carcinoma MDA-MB-231 [24], [25], prompting us to verify whether the H4R agonist CB inhibited their proliferation, which was not the case since at optimal concentrations (10^−5^ M) neither the growth of these cells (**Fig. 7A**) nor their sensitivity to the anti-neoplastic drug 5-FU were affected, as illustrated for the HCT 116 cell line in **Fig. 7B**. However, this result is not conclusive, inasmuch as we were unable to confirm the H4R expression on transcriptional and protein levels reported before (data not shown), which shows that it is indeed essential to assess whether H4R is operational in the cancer cells each time before considering a clinical application.

## Discussion

In this study we provide the first demonstration that the H4R is expressed in murine and human hematopoietic progenitor cells and that its activation results in the inhibition of growth factor-induced cell cycling. In our experimental setup histamine and CB, which is also a H3R antagonist, target the H4R specifically, since 1) bone marrow cells do not express the H3R [5], 2) cell cycle progression was restored when the H4R was blocked by selective H4R antagonists before exposure to CB or histamine and 3) the effect of CB was clearly reduced after H4R silencing. The H4R is functional in hematopoietic progenitor cells since its stimulation inhibited both myeloid and lymphoid colony formation, suggesting a relatively broad distribution among different progenitor subsets identified by their distinct clonogenic potential.

The signal transduction pathway leading from H4R engagement to cell cycle arrest is initiated by G_i/o_ protein coupling followed by a decrease of cAMP levels. Further downstream, it entails downregulation of PKA activity since a specific inhibitor of this enzyme mimicked the effect of CB. Notwithstanding the increased evidence for a crosstalk between cAMP and the ERK/MAPK pathway [22], Erk expression was not modified by exposure to CB. It is therefore most likely that decreased cell cycling is promoted through PKA/CREB signaling, as proposed by others [23].

H4R stimulation resulted in a striking downregulation of cyclin D3, whose expression is typically induced by mitogens [21]. Cyclin E levels were likewise decreased, while CB prevented the growth factor-induced decrease of the cyclin-dependent kinase inhibitor p21^Cip1/Waf1^, a cell cycle regulator with important functions in self-renewal, differentiation and apoptosis of progenitor cells [26]. By contrast, p27^Kip1^ expression that has been associated with the regulation of growth and/or differentiation of more lineage-restricted progenitor cells [27] was decreased in the same conditions. It is currently acknowledged that the suppression of p27^Kip1^ alone is not sufficient to induce hematopoietic progenitor cell cycling [28], as p21^Cip1/Waf1^ presumably compensates for its loss to maintain quiescence [26].

The growth arrest in response to H4R stimulation occurred both *in vitro* and *in vivo*. Indeed, murine progenitor-enriched bone marrow cells recovered after injection of CB were less prompt to enter the cell cycle in response to growth factor cocktail than saline-injected controls. This cell cycle arrest was revealed only when progenitors were induced to proliferate in response to growth factors. Without stimulation, they were mostly quiescent, and thus not affected by H4R activation. The reversibility of the cell cycle arrest, the lack of apoptosis and *in vivo* toxicity qualified H4R agonists as potential pharmacological tools to protect clonogenic cells from the hematotoxicity of anti-cancer drugs, which remains a major drawback of chemotherapy. This toxicity can affect either all hematopoietic lineages or particular subsets, depending on the anti-cancer drug used and the doses administered [29]. Several approaches have been proposed to limit these complications, such as autologous bone marrow transplantation at high-dose chemotherapy [30] or treatment with growth factors like G-CSF or Epo to alleviate neutropenia or to prevent anemia, respectively [31], [32]. Yet, despite their relative effectiveness in accelerating hematopoietic recovery or allowing a more intensive drug regimen, these treatments are expensive, difficult to handle and not devoid of side effects. There is also some evidence that repeated treatment with growth factors during multiple courses of chemotherapy may deplete the stem cell compartment and give rise to genomic alterations, even though these issues remain controversial [33], [34].

The use of negative regulators of hematopoietic progenitor proliferation as a strategy to reduce the hematotoxicity of anti-neoplastic drugs, while improving the anti-tumor activity by dose intensification, has been proposed in previous studies [35]. Some of these compounds have proved quite effective *in vitro* or in murine *in vivo* models [36]–[37], but turned out to be less beneficial in preclinical trials because of a number of side effects [38]. It has also been reported that chemokines act in synergy to decrease the percentage of progenitors in S phase, thus providing myeloprotection against cell cycle-dependent drugs *in vitro* and *in vivo* and accelerating hematopoietic recovery [39]. It has not yet been established whether this effect is direct or indirect and how chemokine receptors are distributed on different hematopoietic progenitor subsets.

Among the multiple biological activities ascribed to histamine, its contribution to the regulation of cell proliferation is supported by a number of reports in normal and tumor cells [40]. These studies have produced numerous and often conflicting results, probably because histamine exerts different effects depending on its binding sites and their distribution on target cells. Although it is generally acknowledged that H4Rs are mainly expressed in hematopoietic cells, some H4R^+^ tumor cell lines and colorectal cancer biopsies have also been reported [24], [25], [41], [42]. We failed to confirm this result in the colon carcinomas, HT29 and HCT116, and the mammary carcinoma MDA-MB-231. In accordance with the lack of expression, we found that the H4R agonist CB affected neither the proliferation of these malignant cells nor did it decrease their sensitivity to *in vitro* treatment with the antineoplastic drug 5-FU, which raises the question of the reliability of results obtained with long-established cell lines.

It is obvious that in a clinical setting H4R expression in cancer cells should be assessed in each patient before treatment and avoided when positive. Note that the studies investigating the effect of H4R activation on cancer cell cycle progression have given rise to conflicting results so far. For example, in MDA-MB-231 cells H4R activation has been shown to result in G0/G1 cell cycle arrest followed by apoptosis [25], while in another report histamine receptor-mediated cell cycle arrest occurred in G2/M, once again followed by apoptosis and enhanced radio-sensitivity, which would increase the therapeutic efficiency rather than protect malignant cells [41]. It has also been proposed that H4R expression is downregulated in colorectal tumour cells [42], as compared with healthy tissue, which underscores once again the requirement of individual tests.

In conclusion, our data provide evidence for a new site of intervention of histamine, through which it can specifically slow down cell cycle progression in hematopoietic progenitors. At this point, we can only speculate on the physiological relevance of this interaction in patho-physiological situations generating increased histamine levels in hematopoietic organs. Nonetheless, this new function of the H4R provides an original therapeutic strategy to alleviate the side effects of chemotherapy by preventing clonogenic progenitors from entering the cell cycle, rendering them less susceptible to the toxicity of anti-cancer drugs. We have not established so far whether the H4R is functional in hematopoietic stem cells with long-term repopulation activity, which in any case would be irrelevant in this particular context, because this quiescent population is not affected by cell cycle-dependent drugs [43]. The purpose of our approach is to limit the period of aplasia by accelerating short-term reconstitution from more lineage-restricted clonogenic progenitors without impairing the sensitivity of cancer cells to the treatment. These clinical perspectives, which might eventually facilitate chemotherapy dose and schedule intensification, call for the development of new, selective H4R agonists.

## Materials and Methods

### Ethics statement

Animal experiments were performed according to the French Institutional Committee.

CD34^+^ cells were purified from cord blood recovered with the parents' written consent, in accordance with the recommendations and approval of the Belgian Ethical Committee (Comité éthique hospitalo-facultaire universitaire Liège 707). The experiments were with frozen purified CD34^+^ cells in the research unit CNRS UMR8147 were performed with the approval of the local Ethics Committee (Comité d'éthique de l'Hôpital Necker).

### Mice

6- to 8-week-old, specific pathogen-free, male or female C57BL/6J mice were purchased from CERJ (Les Genest St. Isle, France) and maintained in our animal facility. All mice were kept in well-controlled animal housing facilities and had free access to tap water and pellet food. Animal experiments were performed according to the French Institutional Committee.

### Cytokines, histamine receptor ligands, antibodies and other compounds

Murine and human recombinant IL-3, IL-6, Flt3-L, GM-CSF and SCF were purchased from R&D Systems (Abingdon, UK). Thioperamide, histamine dihydrochloride, forskolin, dibutyryl (db)cAMP and the PKA inhibitor Rp-8-Br-cAMPS were purchased from Sigma-Aldrich (St.-Quentin Fallavier, France). The H4R agonist clobenpropit dihydrobromide was from Tocris (Ellisville, MO), as was the H4R antagonist JNJ10191585. JNJ7777120 was provided by UCB-Pharma. TrueLabeling-AMP^TM^ Linear RNA Amplification Kit and Oligo GEArray® Mouse cell cycle Microarray (OMM-020) were purchased from SABiosciences (tebu-bio, France) The following appropriately labeled antibodies were used: anti-mouse CD117 (c-kit) (2B8), TCRβ (H57-597), Ly-6G(Gr-1) (RB6-8C5), CD11b (M1/70), CD45R(B220) (RA3-6B2), CD4 (H129.19), CD8 (53–6.7), CD19 (1D3), TER-119, Ly-6A/E (Sca-1) (D7) and anti-human CD34 (581) (all from Pharmingen, San Diego, CA). Polyclonal goat anti-human and anti-mouse H4R Abs, donkey anti-goat-FITC, corresponding blocking peptides and irrelevant control goat Abs were purchased from Santa Cruz Biotechnology (Santa Cruz, CA). FITC-conjugated anti-cyclin D3 and isotype controls were likewise from Santa Cruz. *Bordetella pertussis* toxin was from Calbiochem (La Jolla, CA).

### Cell preparations, FACS analysis and sorting

Bone marrow and spleen cells were recovered and suspended in culture medium, as previously described [5]. Peripheral blood was collected from the retro-orbital sinus and nucleated cells were counted on a MS9-5 Hematology Counter (Melet Schloesing Laboratories, Osny, France). CD34^+^ cells were purified from cord blood recovered with the parents' consent, in accordance with the recommendations of the Belgian Ethical Committee. They were sorted magnetically using the positive selection kit purchased from Miltenyi (Bergisch-Gladbach, FRG).

Cell suspensions were incubated on ice in the presence of rat anti-mouse CD16/CD32 mAb (1 µg/10^6^ cells, Pharmingen) to block Fc receptor functions prior to specific staining with appropriately labeled antibodies. For intracellular labeling with anti-H4R and anti-cyclin D3 antibodies cells were treated with FACS permeabilizing solution from BD before staining Cells were analyzed in a FACSCanto II cytofluorometer (Becton Dickinson, Mountain View, CA), using FlowJo software. Erythrocytes and debris were excluded on the basis of forward and side scatter parameters. At least 10,000 cells were acquired within the live gate.

Lin^−^ cells designate a progenitor-enriched population expressing neither myeloid nor lymphoid lineage markers (negative for CD11b, Gr-1, CD19, TCRβ). Lin^−^ and c-kit^+^ cells (>95% purity) were isolated using the SpinSep depletion kit and the positive selection kit with the RoboSep automaton from StemCell Technologies, respectively. After staining, c-kit^+^Sca1^+^ cells were further sorted electronically using a FACSVantage^TM^ cell sorter (Becton Dickinson). The effect of CB on apoptosis of progenitor-enriched BMC was evaluated using an Annexin-V staining kit (Pharmingen). All these reagents were used according to the manufacturer's instructions.

### Cell cultures

Progenitor-enriched Lin^−^, c-kit^+^ or CD34^+^ cells were suspended at a final concentration of 10^5^ cells per ml in serum-free StemSpan medium (StemCell Technologies) and cultured up to 3 days in the presence of 10 ng of IL-3, 10 ng of IL-6 and 50 ng of SCF per ml, with or without CB (10^−5^ M). They were examined at different time points for cell cycle progression as well as phenotypic and morphologic characteristics, using cytometry and light microscopy analysis of MGG-stained cytospin preparations.

Murine CFU-GM and human CFU-C were quantified in MethoCult M3230 (StemCell Technologies) supplemented with IL-3 alone (1 ng/ml) and in MethoCult GF H4434 (complete with growth factors), respectively. They were plated in a final volume of 1 ml at a concentration of 5×10^4^ total BM cells/culture dish (Falcon 1008) for murine and 5000 CD34^+^ cells/ml for human progenitors. Colonies were scored on day 7 and day 14, respectively. *In vitro* protection against myelotoxic AraC (Cytarabine, Pfizer) and hydroxy-urea (Sigma) was evaluated after a 24-h pretreatment of total BMC or CD34^+^ cells with CB (10^−5^ M), followed by exposure to 10 µg/ml of AraC or 10^−3^ M hydroxy-urea (murine) for 1 h, extensive washing and colony-forming assay.

In some experiments clonogenic progenitors were evaluated in the HALO^TM^-Hemogenix assay (Hemogenix Inc., Colorado Springs, CA) based on luminescence output using adequate growth factor cocktails for CFU-GEMM, CFU-GM and BFU-E colony formation, according to the manufacturer's instructions.

Clonogenic common lymphoid progenitors were evaluated in MethoCult M3230 (StemCell Technologies) supplemented with murine recombinant SCF (100 ng/ml), IL-7 (10 ng/ml and Flt-3-L (20 ng/ml). All cultures were incubated at 37°C in a humidified chamber under 5% CO_2_.

### Tracking of cell divisions and evaluation of cell cycle status

10^5^ Lin^−^ BMC/ml were incubated at 37°C with 5 mM CFSE (Invitrogen) in PBS. After 10 min, a 5-fold excess of ice-cold PBS was added to stop the reaction. Cells were then centrifuged, re-suspended and cultured for 72 h in serum-free StemSpan medium supplemented with IL-3, IL-6 and SCF (10, 10 and 50 ng/ml, respectively, with or without CB (10^−5^ M). Thereafter, they were labeled with APC-conjugated anti-c-kit antibody and CFSE fluorescence was analyzed in c-kit^+^ cells.

After incubation, 10^5^ cells were labeled with FITC-conjugated c-kit antibody, fixed in PBS 1% formaldehyde-free methanol that was removed before treatment with 1 ml of PI/Triton X100 (20 µg/ml/0.1%)+DNase-free RNase (0.2 µg/ml) for 30 min at room temperature. In some experiments magnetically sorted c-kit^+^ BMC were incubated for 2 h in StemSpan medium with growth factor cocktail alone or in the presence of CB (10^−5^ M), dbcAMP (5×10^−4^ M), forskolin (10^−5^ M) with or without CB. The cell cycle status was then assessed using Vybrant DyeCycle Violet (VDV) Stain, according to the manufacturer's instructions. The effect of *Bordetella pertussis* toxin (100 ng/ml) on the cell cycle arrest promoted by CB was evaluated in the same conditions. After incubation, cells were analyzed using FlowJo sofware.

### *In vivo* experiments

The cell cycle status of bone marrow CFU-C was analyzed after three i.p. injections of CB at a concentration of 30 mg/kg each at 24, 16 and 4 h before sacrifice. Bone marrow cells were then recovered, incubated overnight at a concentration of 10^6^ cells/ml in growth factor cocktail (10 ng of IL-3, 10 ng of IL-6 and 50 ng of SCF per ml) followed by PI staining and cell cycle analysis among gated Lin^−^ cells.

The myeloprotective effect of CB-induced cell cycle arrest was assessed after treatment with AraC or cyclophosphamide (Endoxan: Baxter, France). Mice received three i.p. injections of CB at a dose of 20 mg/kg, at 24 and 4 h before, as well as 24 h after chemotherapy. AraC and cyclophosphamide were given i.p. at a dose of 350 mg/kg and 300 mg/kg, respectively. Mice were sacrificed on day 2 post-chemotherapy, when white blood cells were counted and colony-forming cells were evaluated in methylcellulose in response to IL-3.

### Western blotting, cell cycle microarray and H4R silencing

Whole-cell extracts for Western blot analysis were prepared from freshly sorted c-kit^+^ cells after a 5-h incubation in StemSpan medium with growth factors alone or together with CB. 30 µg extracts were prepared from washed cells in 100 µl extraction buffer. They were subjected to SDS-PAGE and transferred onto nitrocellulose sheets (Hybond C, Amersham Biosciences). Protein transfer was ascertained by Ponceau red coloration. Membranes were then washed with PBS and blocked with PBS-Tween 0.1%-gelatin 0.5% for 1 h at room temperature. Blotting with monoclonal anti-cyclin E antibodies (Santa Cruz Biotechnology) was performed according to the manufacturer's instructions. The same membranes were then stripped and reprobed with monoclonal antibodies against p21^Waf1/Cip^, p-Erk1/2, Erk 1/2, actin (Santa Cruz Biotechnology) or p27^Kip1^ (Pharmingen). Oligo GEArray® assays were performed with freshly isolated sorted c-kit^+^ BM cells or after a 3-h incubation in the presence of growth factor cocktail with or without CB. RNA was extracted and amplified with the TrueLabeling-AMP^TM^ Linear RNA Amplification kit according to the manufacturer's instructions. The array OMM-20 contains 128 oligonucleotide probes representing genes associated with the cell cycle. H4R silencing was performed with freshly sorted c-kit^+^ cells and expanded CD34^+^ cells. For transfection, 3 µl of HiPerFect reagent (Qiagen; Courtaboeuf, France) and 4 µl (40 pmol) of human and 8 µl (80 pmol) of murine H4R siRNA, scrambled FITC-labeled or unconjugated control siRNAs (all from Santa Cruz Biotechnology) were incubated in 50 µl x-vivo medium (Cambrex; East Rutherford, NJ) for 20 min at room temperature. The mixture was then added to 50 µl of cell suspension at a final concentration of 10^6^ cells/ml and incubated for 20 h, when transfection efficiency was verified with scrambled FITC-conjugated siRNA. Cells were then centrifuged and resuspended at a concentration of 10^5^ cells/ml in StemSpan medium with growth factor cocktail for c-kit^+^ and CD34^+^ cells. The extinction of H4R protein expression was ascertained by FACS analysis after 24 h. After 3 days of culture with or without CB, cells were counted and their viability was assessed by trypan blue exclusion. The cell cycle status was assessed after an overnight incubation.

### Statistics

Statistical significance was established by Student's and Mann-Whitney's two-tailed t test.

## Supporting Information

Figure S1 Dose response curve of cell cycle arrest induced by CB.
(6.01 MB TIF)

Figure S2 Apoptosis was evaluated by Annexin-V/PI staining after a 3-day incubation of progenitor-enriched Lin- BMC in the presence of growth factor cocktail, with or without CB (10–5 M). A typical experiment is depicted.
(6.01 MB TIF)

Figure S3 Blockade of H4R by specific antagonists abrogates CB-induced cell cycle arrest. Cell cycle was analyzed after VDV staining in sorted progenitor-enriched c-kit+ BM cells after a 2-h incubation in StemSpan medium supplemented growth factor cocktail (GF), with or without CB at a concentration of 10–5 M. The H4R antagonists were added at a concentration of 10–5 M 10 min before CB.
(6.01 MB TIF)

## Acknowledgements
