# Supplementary figures and images for: H4 Histamine Receptors Mediate Cell Cycle Arrest in Growth Factor-Induced Murine and Human Hematopoietic Progenitor Cells
Source: PLoS One. 2009 Aug 7;4(8):e6504. doi: 10.1371/journal.pone.0006504 (PMC2720606; doi:10.1371/journal.pone.0006504)

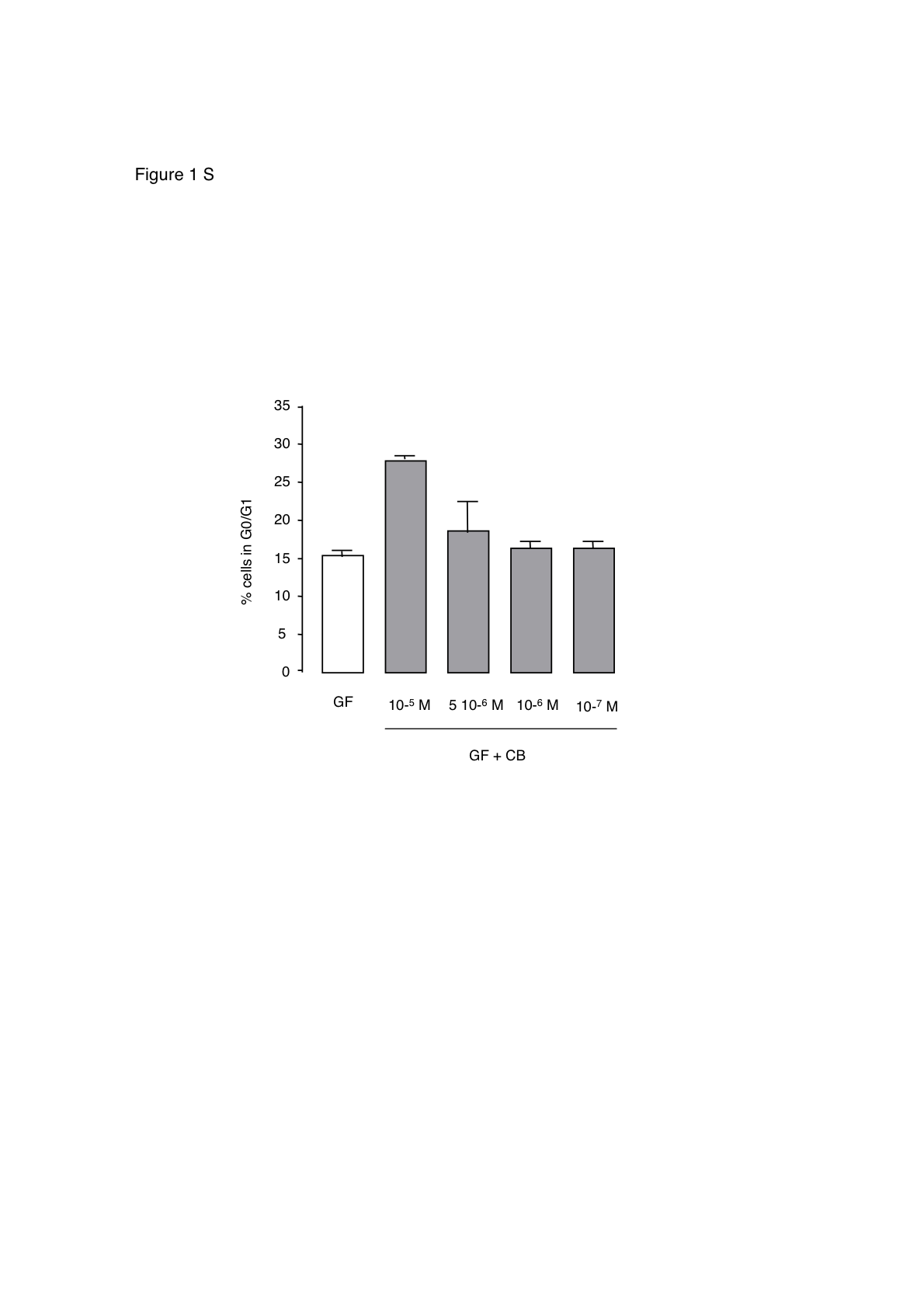

Supplement: Figure S1 — Dose response curve of cell cycle arrest induced by CB. (6.01 MB TIF) [file pone.0006504.s001.tif]

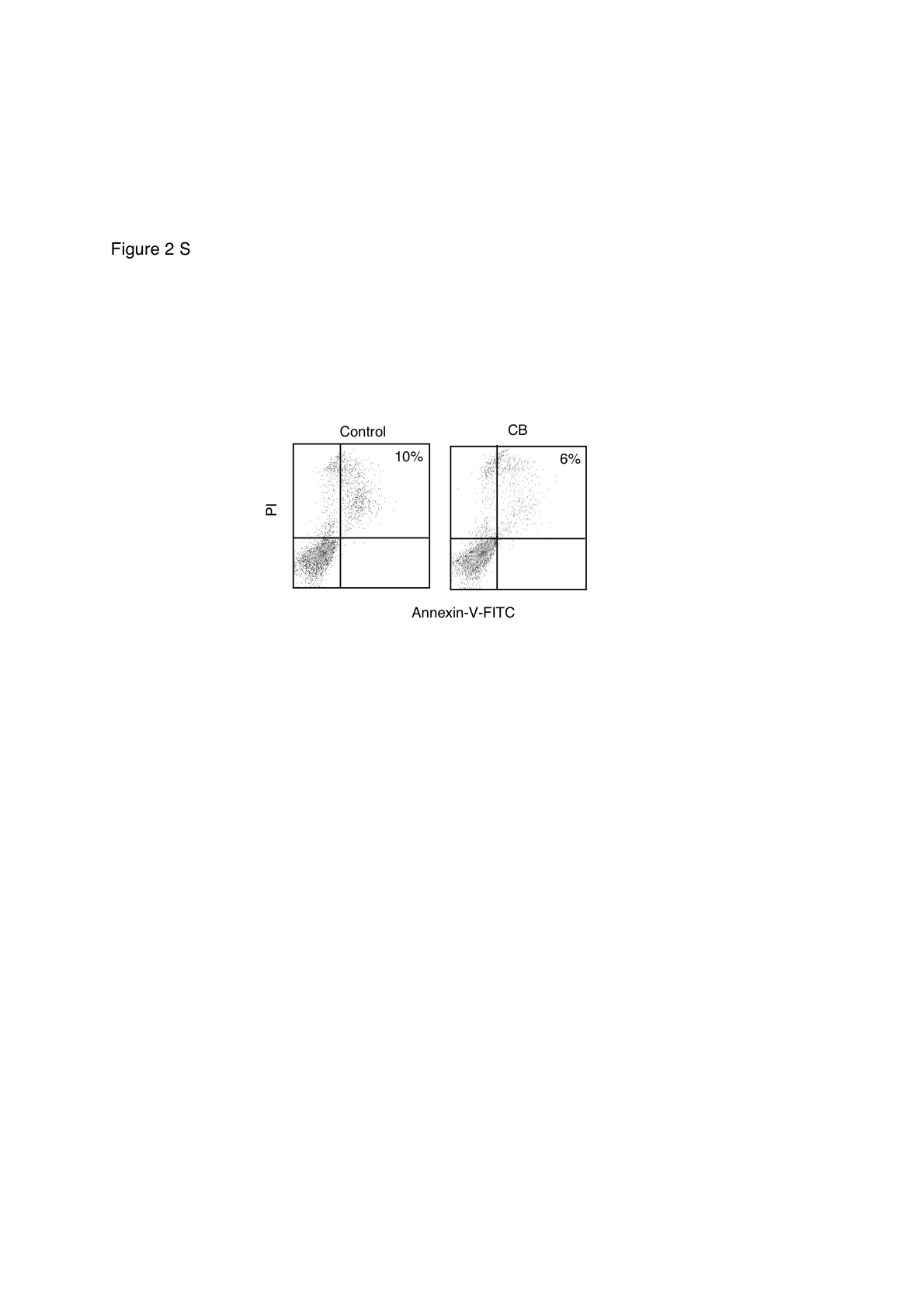

Supplement: Figure S2 — Apoptosis was evaluated by Annexin-V/PI staining after a 3-day incubation of progenitor-enriched Lin- BMC in the presence of growth factor cocktail, with or without CB (10–5 M). A typical experiment is depicted. (6.01 MB TIF) [file pone.0006504.s002.tif]

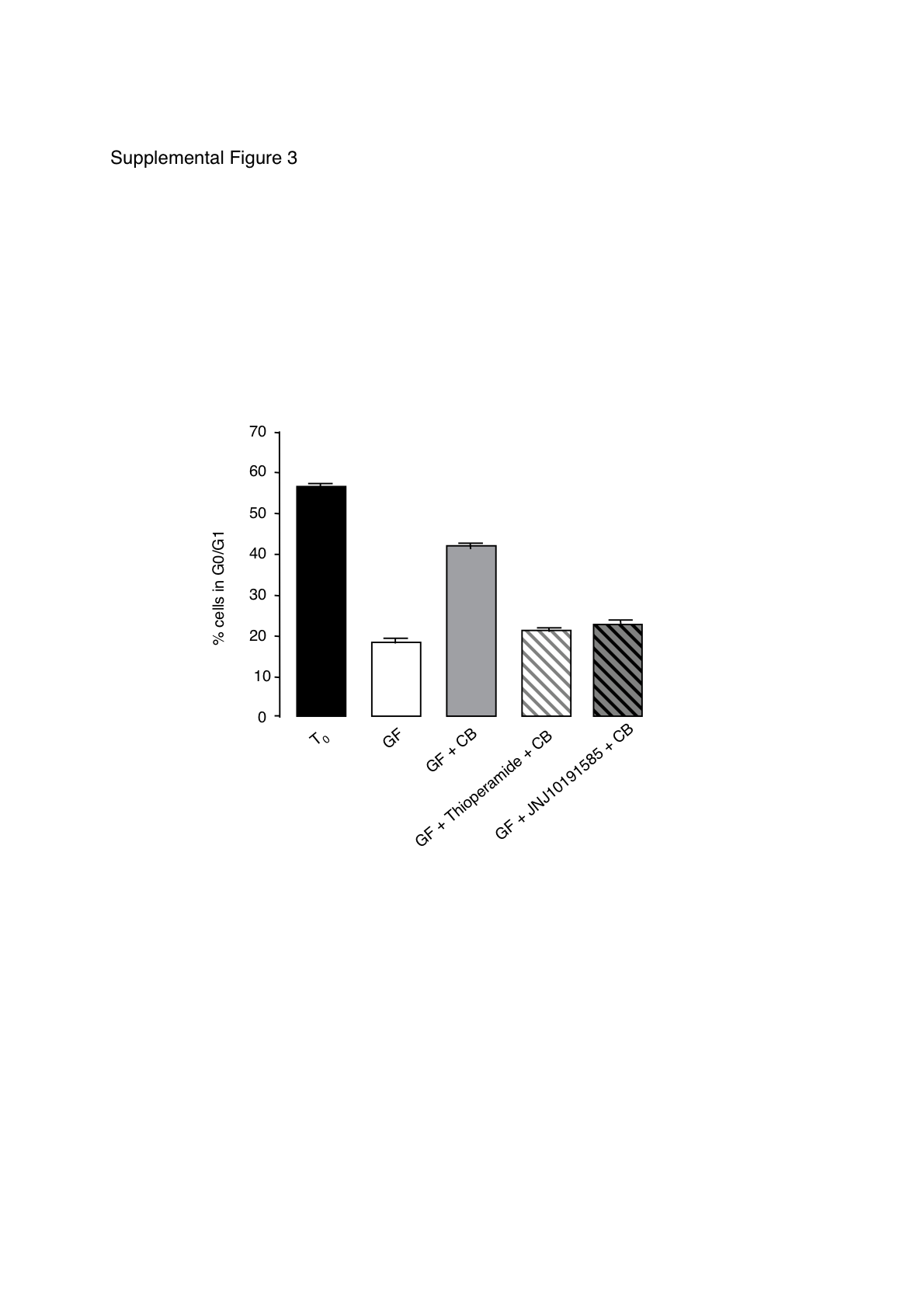

Supplement: Figure S3 — Blockade of H4R by specific antagonists abrogates CB-induced cell cycle arrest. Cell cycle was analyzed after VDV staining in sorted progenitor-enriched c-kit+ BM cells after a 2-h incubation in StemSpan medium supplemented growth factor cocktail (GF), with or without CB at a concentration of 10–5 M. The H4R antagonists were added at a concentration of 10–5 M 10 min before CB. (6.01 MB TIF) [file pone.0006504.s003.tif]
